# Poly[(μ_2_-azido-κ^2^ *N* ^1^:*N* ^1^)[μ_2_-5-(8-quinolyloxymeth­yl)tetra­zolato-κ^4^ *N* ^1^,*O*,*N* ^5^:*N* ^4^]manganese(II)]

**DOI:** 10.1107/S1600536808022617

**Published:** 2008-07-23

**Authors:** Fang Chen, Heng-Yun Ye

**Affiliations:** a Ordered Matter Science Research Center, College of Chemistry and Chemical Engineering, Southeast University, Nanjing 210096, People’s Republic of China

## Abstract

In the structure of the title compound, [Mn(C_11_H_8_N_5_O)(N_3_)]_*n*_, the Mn atoms are hexa­coordinated by five N atoms and one O atom. The coordination polyhedron of the Mn atom is a slightly distorted octa­hedron. The Mn atoms are connected by azide anions with a μ_2_-1,1 mode and by 5-(8-quinolyloxymeth­yl)tetra­zolate ligands in a μ_2_-η^1^(*N*),η^3^-(*N,N,O*) fashion to form a two-dimensional framework parallel to the (100) plane. Geometric parameters of the organic ligand are in the normal ranges and the dihedral angle between the quinoline ring system and the tetra­zole unit is 7.41 (15)°. The structure involves intra- and inter­molecular C—H⋯N hydrogen bonds.

## Related literature

For the use of tetra­zole derivatives in coordination chemistry, see: Wang *et al.* (2005 ▶); Xiong *et al.* (2002 ▶). For the crystal structure of a tetra­zole derivative, see: Wang & Ye (2007 ▶); For the synthesis of 8-cyanato­quinoline, see: Luo & Ye (2008 ▶).
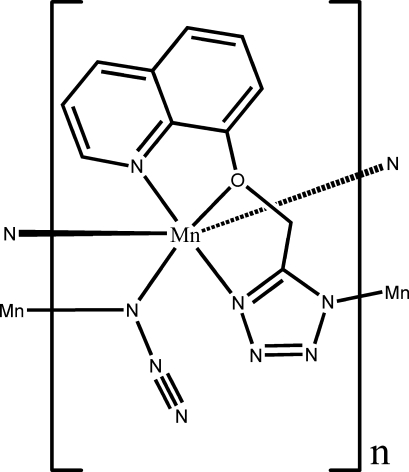


## Experimental

### 

#### Crystal data

[Mn(C_11_H_8_N_5_O)(N_3_)]
*M* *_r_* = 323.19
Monoclinic, 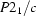

*a* = 10.431 (2) Å
*b* = 14.431 (3) Å
*c* = 8.589 (2) Å
β = 90.676 (18)°
*V* = 1292.8 (5) Å^3^
*Z* = 4
Mo *K*α radiation
μ = 1.03 mm^−1^
*T* = 293 (2) K
0.20 × 0.16 × 0.12 mm

#### Data collection

Rigaku, SCXmini diffractometer
Absorption correction: multi-scan (*CrystalClear*; Rigaku, 2005 ▶) *T*_min_ = 0.820, *T*_max_ = 0.886
13382 measured reflections
3074 independent reflections
2472 reflections with *I* > 2σ(*I*)
*R*_int_ = 0.054

#### Refinement

*R*[*F* ^2^ > 2σ(*F* ^2^)] = 0.046
*wR*(*F* ^2^) = 0.105
*S* = 1.11
3074 reflections
190 parameters
H-atom parameters constrained
Δρ_max_ = 0.32 e Å^−3^
Δρ_min_ = −0.41 e Å^−3^

### 

Data collection: *CrystalClear* (Rigaku, 2005 ▶); cell refinement: *CrystalClear*; data reduction: *CrystalClear*; program(s) used to solve structure: *SHELXS97* (Sheldrick, 2008 ▶); program(s) used to refine structure: *SHELXL97* (Sheldrick, 2008 ▶); molecular graphics: *SHELXTL* (Sheldrick, 2008 ▶); software used to prepare material for publication: *SHELXTL*.

## Supplementary Material

Crystal structure: contains datablocks I, global. DOI: 10.1107/S1600536808022617/rk2100sup1.cif

Structure factors: contains datablocks I. DOI: 10.1107/S1600536808022617/rk2100Isup2.hkl

Additional supplementary materials:  crystallographic information; 3D view; checkCIF report

## Figures and Tables

**Table 1:** Hydrogen-bond geometry (Å, °)

*D*—H⋯*A*	*D*—H	H⋯*A*	*D*⋯*A*	*D*—H⋯*A*
C2—H2*B*⋯N8^i^	0.97	2.50	3.223 (4)	131
C5—H5*A*⋯N3^ii^	0.93	2.49	3.413 (4)	173

Symmetry codes: (i) 
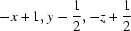
; (ii) 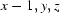.

**Fig. 1.**
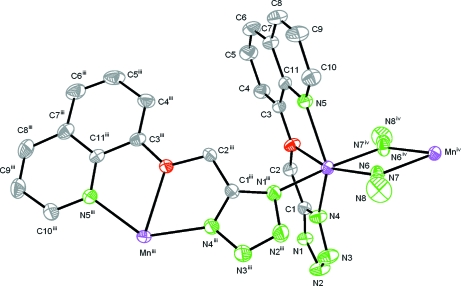
The fragment structure of I showing the atom numbering scheme. Displacement ellipsoids are drawn at the 30% probability level. The hydrogen atoms are presented as small spheres of arbitrary radius. Symmetry codes: (iii) 1-x, 2-y, -z; (iv) x, 3/2-y, 1/2+z.

**Fig. 2.**
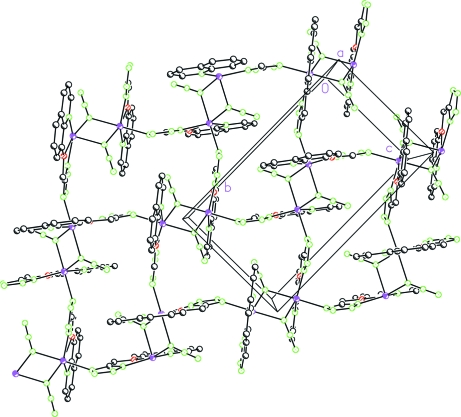
Two–dimensional net framework of the title compound view along a axis and all hydrogen atoms are omitted for clarity.

**Fig. 3.**
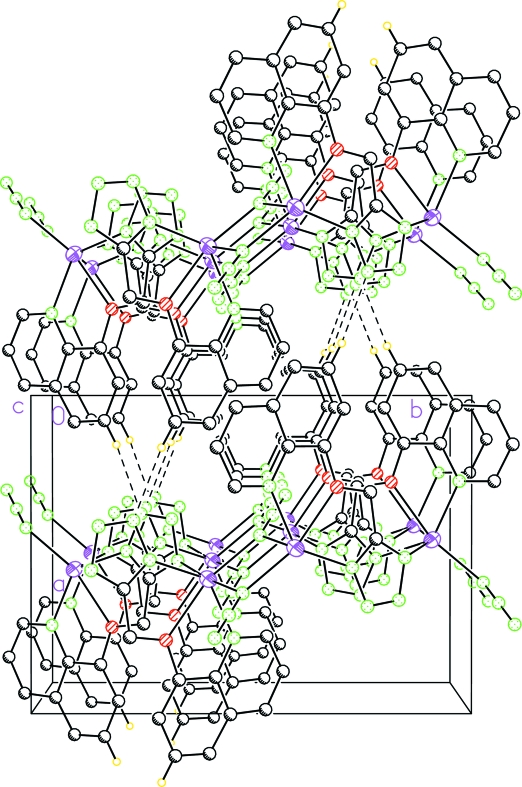
Crystal packing of the title compound viewed along the c axis. Hydrogen atoms not included in intermolecular hydrogen bonds are omitted for clarity. Dashed lines show the intermolecular hydrogen bonds.

## supplementary crystallographic information

### Comment

In the past five years, we have focused on the chemistry of 5–substituted tetrazole because of their multiple coordination modes as ligand to metal ions and the construction of novel metal–organic framework (Wang *et al.* 2005; Xiong *et al.* 2002). As part of our on going studies of the chemistry of tetrazole, we determined the crystal structure of the title compound, *catena*–[(*µ*_2_–1,1–azido)–(*µ*_2_– *η*^1^(*N*), *η*^3^–(*N,N,O*)–((tetrazol–5–yl)methoxy) quinoline)–Manganese], I (Fig. 1).

As shown in Fig. 1, Mn1 is hexa–coordinated by five N and one O atoms, of which two N atoms and one O atom are from one organic ligand (tetrazol–5–yl)methoxy–quinoline, one N atom is from the tetrazole unit of another symmetry–related organic ligand (symmetry code: (iv) *x*, 3/2-*y*, 1/2+*z*) and two N atoms are from two azido anions which are symmetry–related (symmetry code: (iii) 1-*x*, 2-*y*, -*z*). The coordinated geometry of Mn1 is a distorted octahedron. The N4, N5, O1  and N6 atoms form the equatorial plane with mean deviation 0.1615Å of the plane (N4, N5, N6, O1 and Mn1). Geometry parameters of organic ligand are in normal ranges (Wang & Ye, 2007), dihedral angle of quinoline unit and the tetrazole unit is 7.41 (15)°. The Mn atoms are connected by azido anions and by (tetrazol–5–yl)methoxy–quinoline) ligands to form two–dimensional net framework parallel to the (1 0 0) plane (Fig.2). Beside the van der Waals forces, the crystal structure of I is also stabilized by intermolecular C—H···N^ii^ hydrogen bonds. Symmetry code: (ii) *x*-1, *y*, *z* (Fig. 3).

### Experimental

The precusor organic compound 8–cyanatoquinoline is was synthesized by using a similar procedure described by us before (Luo & Ye, 2008).A mixture of the organic ligand (34 mg, 0.2 mmol), NaN_3_ (20 mg, 0.3 mmol), MnCl_2_(25 mg, 0.2 mmol) and water (1 ml) sealed in a glass tube was maintained at 423 K. Yellow crystals suitable for X–ray analysis were obtained after 2 days.

### Refinement

All H atoms were positioned geometrically and refined using a riding model with d(C—H)_methine_ = 0.98Å, d(C—H)_aryl_ = 0.93Å, *U*_iso_ = 1.2*U*_eq_(C).

### Crystal data

**Table:**

[Mn(C_11_H_8_N_5_O)(N_3_)]	*F*_000_ = 652
*M**_r_* = 323.19	*D*_x_ = 1.661 Mg m^−^^3^
Monoclinic, *P*2_1_/*c*	Mo *K*α radiation λ = 0.71073 Å
Hall symbol: -P 2ybc	Cell parameters from 3074 reflections
*a* = 10.431 (2) Å	θ = 2.8–27.9º
*b* = 14.431 (3) Å	µ = 1.03 mm^−^^1^
*c* = 8.589 (2) Å	*T* = 293 (2) K
β = 90.676 (18)º	Block, yellow
*V* = 1292.8 (5) Å^3^	0.20 × 0.16 × 0.12 mm
*Z* = 4	

### Data collection

**Table:**

Rigaku, SCXmini diffractometer	3074 independent reflections
Radiation source: Fine-focus sealed tube	2472 reflections with *I* > 2σ(*I*)
Monochromator: Graphite	*R*_int_ = 0.054
Detector resolution: 13.6612 pixels mm^-1^	θ_max_ = 27.9º
*T* = 293(2) K	θ_min_ = 2.8º
ω scans	*h* = −13→13
Absorption correction: multi-scan(CrystalClear; Rigaku, 2005)	*k* = −19→19
*T*_min_ = 0.820, *T*_max_ = 0.886	*l* = −11→11
13382 measured reflections	

### Refinement

**Table:**

Refinement on *F*^2^	Secondary atom site location: difference Fourier map
Least-squares matrix: full	Hydrogen site location: inferred from neighbouring sites
*R*[*F*^2^ > 2σ(*F*^2^)] = 0.046	H-atom parameters constrained
*wR*(*F*^2^) = 0.106	*w* = 1/[σ^2^(*F*_o_^2^) + (0.0431*P*)^2^ + 0.2319*P*] where *P* = (*F*_o_^2^ + 2*F*_c_^2^)/3
*S* = 1.11	(Δ/σ)_max_ = 0.001
3074 reflections	Δρ_max_ = 0.32 e Å^−^^3^
190 parameters	Δρ_min_ = −0.41 e Å^−^^3^
Primary atom site location: structure-invariant direct methods	Extinction correction: None

### Special details

**Table:**

Geometry. All s.u.'s (except the s.u. in the dihedral angle between two l.s. planes) are estimated using the full covariance matrix. The cell s.u.'s are taken into account individually in the estimation of s.u.'s in distances, angles and torsion angles; correlations between s.u.'s in cell parameters are only used when they are defined by crystal symmetry. An approximate (isotropic) treatment of cell s.u.'s is used for estimating s.u.'s involving l.s. planes.
Refinement. Refinement of *F*^2^ against ALL reflections. The weighted *R*–factor *wR* and goodness of fit *S* are based on *F*^2^, conventional *R*–factors *R* are based on *F*, with *F* set to zero for negative *F*^2^. The threshold expression of *F*^2^ > σ(*F*^2^) is used only for calculating *R*–factors(gt) *etc*. and is not relevant to the choice of reflections for refinement. *R*–factors based on *F*^2^ are statistically about twice as large as those based on *F*, and *R*–factors based on ALL data will be even larger.

### Fractional atomic coordinates and isotropic or equivalent isotropic displacement parameters (Å^2^)

**Table:**

	*x*	*y*	*z*	*U*_iso_*/*U*_eq_	
Mn1	0.44828 (3)	0.90619 (3)	0.09904 (4)	0.03138 (13)	
C1	0.4312 (2)	0.72913 (17)	−0.1025 (3)	0.0347 (6)	
C2	0.2897 (2)	0.74195 (18)	−0.0956 (3)	0.0361 (6)	
H2A	0.2548	0.7575	−0.1974	0.043*	
H2B	0.2484	0.6860	−0.0588	0.043*	
C3	0.1497 (2)	0.84401 (18)	0.0504 (3)	0.0369 (6)	
C4	0.0389 (3)	0.8016 (2)	0.0034 (4)	0.0489 (7)	
H4A	0.0410	0.7513	−0.0642	0.059*	
C5	−0.0791 (3)	0.8355 (2)	0.0595 (5)	0.0636 (9)	
H5A	−0.1549	0.8068	0.0280	0.076*	
C6	−0.0843 (3)	0.9086 (2)	0.1577 (5)	0.0611 (9)	
H6A	−0.1631	0.9291	0.1939	0.073*	
C7	0.0296 (3)	0.9541 (2)	0.2056 (4)	0.0476 (7)	
C8	0.0335 (3)	1.0319 (2)	0.3054 (4)	0.0598 (9)	
H8A	−0.0425	1.0565	0.3429	0.072*	
C9	0.1473 (3)	1.0708 (2)	0.3471 (4)	0.0622 (9)	
H9A	0.1498	1.1213	0.4144	0.075*	
C10	0.2607 (3)	1.0341 (2)	0.2875 (3)	0.0506 (7)	
H10A	0.3380	1.0617	0.3161	0.061*	
C11	0.1485 (2)	0.92186 (18)	0.1507 (3)	0.0350 (6)	
N1	0.4916 (2)	0.66357 (15)	−0.1804 (3)	0.0402 (5)	
N2	0.6184 (2)	0.67775 (19)	−0.1497 (3)	0.0551 (7)	
N3	0.6309 (2)	0.74859 (19)	−0.0569 (3)	0.0569 (7)	
N4	0.5125 (2)	0.78285 (16)	−0.0251 (3)	0.0431 (6)	
N5	0.2634 (2)	0.96208 (15)	0.1926 (2)	0.0372 (5)	
N6	0.5787 (2)	1.01876 (16)	0.1316 (3)	0.0417 (5)	
N7	0.6346 (2)	1.04671 (15)	0.2407 (3)	0.0415 (5)	
N8	0.6923 (3)	1.0756 (2)	0.3458 (3)	0.0701 (9)	
O1	0.27145 (16)	0.81654 (12)	0.0115 (2)	0.0398 (4)	

### Atomic displacement parameters (Å^2^)

**Table:**

	*U*^11^	*U*^22^	*U*^33^	*U*^12^	*U*^13^	*U*^23^
Mn1	0.0286 (2)	0.0303 (2)	0.0353 (2)	−0.00417 (15)	0.00011 (15)	0.00057 (15)
C1	0.0352 (14)	0.0325 (13)	0.0364 (13)	−0.0005 (10)	−0.0019 (11)	−0.0022 (10)
C2	0.0345 (14)	0.0334 (13)	0.0403 (14)	−0.0042 (11)	−0.0015 (11)	−0.0079 (11)
C3	0.0272 (12)	0.0390 (14)	0.0445 (15)	0.0012 (10)	0.0001 (11)	0.0068 (11)
C4	0.0340 (15)	0.0462 (17)	0.067 (2)	−0.0052 (12)	−0.0026 (13)	−0.0041 (14)
C5	0.0276 (15)	0.061 (2)	0.102 (3)	−0.0051 (14)	−0.0040 (16)	0.004 (2)
C6	0.0306 (15)	0.067 (2)	0.086 (3)	0.0049 (14)	0.0069 (16)	0.0028 (18)
C7	0.0363 (15)	0.0509 (17)	0.0558 (18)	0.0087 (13)	0.0078 (13)	0.0063 (14)
C8	0.0510 (19)	0.063 (2)	0.066 (2)	0.0169 (16)	0.0150 (16)	−0.0072 (17)
C9	0.057 (2)	0.063 (2)	0.066 (2)	0.0136 (17)	0.0090 (17)	−0.0211 (17)
C10	0.0492 (18)	0.0498 (18)	0.0529 (18)	−0.0006 (14)	0.0013 (14)	−0.0121 (14)
C11	0.0291 (13)	0.0397 (14)	0.0362 (14)	0.0020 (10)	0.0031 (10)	0.0072 (10)
N1	0.0336 (12)	0.0408 (13)	0.0461 (13)	0.0030 (9)	−0.0019 (10)	−0.0086 (10)
N2	0.0372 (13)	0.0645 (17)	0.0633 (17)	0.0066 (12)	−0.0057 (12)	−0.0228 (13)
N3	0.0349 (13)	0.0648 (17)	0.0708 (18)	0.0001 (12)	−0.0065 (12)	−0.0225 (14)
N4	0.0322 (12)	0.0428 (13)	0.0541 (14)	0.0026 (10)	−0.0034 (10)	−0.0125 (11)
N5	0.0346 (11)	0.0379 (12)	0.0392 (12)	0.0015 (9)	0.0021 (9)	−0.0013 (9)
N6	0.0456 (13)	0.0389 (12)	0.0405 (13)	−0.0140 (10)	−0.0067 (10)	0.0060 (10)
N7	0.0433 (13)	0.0348 (12)	0.0464 (14)	−0.0020 (10)	0.0019 (11)	0.0010 (10)
N8	0.083 (2)	0.073 (2)	0.0529 (17)	−0.0068 (16)	−0.0253 (16)	−0.0143 (14)
O1	0.0290 (9)	0.0403 (10)	0.0501 (11)	−0.0026 (8)	0.0035 (8)	−0.0124 (8)

### Geometric parameters (Å, °)

**Table:**

Mn1—N6	2.135 (2)	C6—C7	1.415 (4)
Mn1—N4	2.184 (2)	C6—H6A	0.9300
Mn1—N1^i^	2.188 (2)	C7—C11	1.411 (4)
Mn1—N5	2.247 (2)	C7—C8	1.412 (4)
Mn1—N6^ii^	2.272 (2)	C8—C9	1.358 (5)
Mn1—O1	2.3682 (18)	C8—H8A	0.9300
C1—N4	1.322 (3)	C9—C10	1.399 (4)
C1—N1	1.322 (3)	C9—H9A	0.9300
C1—C2	1.490 (4)	C10—N5	1.322 (3)
C2—O1	1.430 (3)	C10—H10A	0.9300
C2—H2A	0.9700	C11—N5	1.376 (3)
C2—H2B	0.9700	N1—N2	1.361 (3)
C3—C4	1.364 (4)	N1—Mn1^iii^	2.188 (2)
C3—O1	1.376 (3)	N2—N3	1.302 (3)
C3—C11	1.416 (4)	N3—N4	1.361 (3)
C4—C5	1.414 (4)	N6—N7	1.169 (3)
C4—H4A	0.9300	N6—Mn1^ii^	2.272 (2)
C5—C6	1.353 (5)	N7—N8	1.157 (3)
C5—H5A	0.9300		
			
N6—Mn1—N4	119.03 (9)	C7—C6—H6A	119.9
N6—Mn1—N1^i^	96.42 (8)	C11—C7—C8	116.5 (3)
N4—Mn1—N1^i^	89.22 (9)	C11—C7—C6	119.2 (3)
N6—Mn1—N5	103.19 (9)	C8—C7—C6	124.3 (3)
N4—Mn1—N5	137.42 (8)	C9—C8—C7	120.5 (3)
N1^i^—Mn1—N5	91.43 (8)	C9—C8—H8A	119.7
N6—Mn1—N6^ii^	79.85 (9)	C7—C8—H8A	119.7
N4—Mn1—N6^ii^	89.90 (9)	C8—C9—C10	119.1 (3)
N1^i^—Mn1—N6^ii^	175.15 (8)	C8—C9—H9A	120.4
N5—Mn1—N6^ii^	92.44 (9)	C10—C9—H9A	120.4
N6—Mn1—O1	161.79 (8)	N5—C10—C9	123.2 (3)
N4—Mn1—O1	69.03 (7)	N5—C10—H10A	118.4
N1^i^—Mn1—O1	100.11 (8)	C9—C10—H10A	118.4
N5—Mn1—O1	68.97 (7)	N5—C11—C7	122.7 (3)
N6^ii^—Mn1—O1	84.01 (7)	N5—C11—C3	118.7 (2)
N4—C1—N1	111.6 (2)	C7—C11—C3	118.6 (3)
N4—C1—C2	122.5 (2)	C1—N1—N2	105.2 (2)
N1—C1—C2	125.9 (2)	C1—N1—Mn1^iii^	132.27 (18)
O1—C2—C1	104.99 (19)	N2—N1—Mn1^iii^	115.27 (17)
O1—C2—H2A	110.7	N3—N2—N1	109.1 (2)
C1—C2—H2A	110.7	N2—N3—N4	108.8 (2)
O1—C2—H2B	110.7	C1—N4—N3	105.3 (2)
C1—C2—H2B	110.7	C1—N4—Mn1	121.64 (17)
H2A—C2—H2B	108.8	N3—N4—Mn1	132.69 (18)
C4—C3—O1	125.4 (3)	C10—N5—C11	117.9 (2)
C4—C3—C11	121.5 (2)	C10—N5—Mn1	121.83 (19)
O1—C3—C11	113.0 (2)	C11—N5—Mn1	120.26 (17)
C3—C4—C5	118.8 (3)	N7—N6—Mn1	132.73 (19)
C3—C4—H4A	120.6	N7—N6—Mn1^ii^	126.11 (18)
C5—C4—H4A	120.6	Mn1—N6—Mn1^ii^	100.15 (9)
C6—C5—C4	121.6 (3)	N8—N7—N6	178.1 (3)
C6—C5—H5A	119.2	C3—O1—C2	120.2 (2)
C4—C5—H5A	119.2	C3—O1—Mn1	118.92 (15)
C5—C6—C7	120.2 (3)	C2—O1—Mn1	120.41 (14)
C5—C6—H6A	119.9		

Symmetry codes: (i) *x*, −*y*+3/2, *z*+1/2; (ii) −*x*+1, −*y*+2, −*z*; (iii) *x*, −*y*+3/2, *z*−1/2.

### Hydrogen-bond geometry (Å, °)

**Table:**

*D*—H···*A*	*D*—H	H···*A*	*D*···*A*	*D*—H···*A*
C2—H2B···N8^iv^	0.97	2.50	3.223 (4)	131
C5—H5A···N3^v^	0.93	2.49	3.413 (4)	173

Symmetry codes: (iv) −*x*+1, *y*−1/2, −*z*+1/2; (v) *x*−1, *y*, *z*.
